# Rectifications in the Practice of Auscultation and Percussion

**Published:** 1842-05

**Authors:** Joseph Skoda

**Affiliations:** Teacher of Practical Medicine in the Hospital of Vienna


*Rectifications in the Practice of Auscultation and Percussion.* By Dr. Joseph Skoda, Teacher of Practical Medicine in the Hospital of Vienna.—The following observations on auscultation, extracted from the lectures of Dr. Skoda, of Vienna, by Dr. Drysdale and Dr. Russell, will be interesting to the English reader; as they place in a new light many facts which we have been accustomed to view differently. They have, moreover, been revised by Dr. Skoda himself.

All will acknowledge, that as the voice is produced in the larynx, it must invariably be transmitted thence to the different parts of the chest; and at first sight it would appear that the common opinion entertained by the profession is correct, namely, that its greater or less distinctness is owing to the good or bad conducting power of the parts lying between the larynx and the parietes of the chest. The correctness of this opinion, however, is very doubtful. For example, in pneumonia, when there is hepatization of the lung, the voice is sometimes distinct, and at other times very indistinct, while percussion shows that no particular change has taken place.

The cause of the occasional disappearance of the resonance of the voice is the obstruction by fluid matter of the bronchial tubes of the hepatized portion of the lung; for the resonance reappears readily when the patient makes a deep inspiration or coughs. This disappearance and return of the resonance, while in other essential particulars the hepatization remains the same, does not accord with the commonly assigned cause; for, according to it, it would be a matter of indifference whether the bronchial tubes contained air or not. In pleuritic effusion into the cavity of the chest, the intensity of the resonance of the voice diminishes as the quantity of the exudation increases; while the contrary should happen if the increased distinctness of the voice at any stage of the effusion depended on the superior conducting power of the interposed fluid.

The question of the superiority in conducting power of dense over rare bodies, has been too much regarded as an abstract law, without paying sufficient attention to the particular circumstances which may modify or prevent its operation. It is quite true that dense bodies conduct the sound more readily than rare ones, but only if the sound be confined to the medium in which it is formed, for it passes with difficulty from one medium to another. For example, the slightest scratching at the end of a long pole is heard distinctly when the other end is placed in contact with the ear, while, if this be not done, (*i. e.* if the sound be transmitted by the air), nothing at all is heard. The striking together of two stones under water, when the head is immersed, is distinctly heard, while no sound is audible when it is taken out. On the other hand the human voice, which is formed in the air, is heard furthest in that medium. When the head is dipped into water, sounds produced in the air are heard very faintly or not at all; and solid substances, as a board or a wall, interrupt sounds more or less completely. The laws of physics teach us further, that sound is more or less reflected in its transmission from a rare medium to a denser one, and that the new medium takes up less than would have been propagated in the same space had it remained in the medium by which it had been till then transmitted; and the less sound is taken up by the new medium, the greater the difference of consistence and coherence between the two media. The reason why enclosed passages and tubes, whose walls are of solid materials, conduct sounds better than the open air is, because they reflect the vibrations which are thus confined to a small space, and prevented from being dispersed and lost in the surrounding air. If the walls of the tube were instrumental in conducting the sound, it is singular that a hollow tube should be used as a stethoscope, and not a solid cylinder of wood or metal. The voice, therefore, reaches the parenchyma of the lungs, not through the solid parts, but through the air in the trachea and bronchia, and ought to be carried further in the healthy lung, in which the air penetrates, into the air-cells, than in the hepatized lung, where the air-cells and smaller bronchia are obliterated. These vibrations, likewise, should pass more easily from the ear into the light tissue of the healthy lung than to the condensed parenchyma of the hepatized one, according to the law explained above.

A consideration of these facts would be almost sufficient in themselves to prevent us from acquiescing in the ordinary opinion, that the reason of the voice being louder when the lung is hepatized, than when it is sound and spongy, depends upon its being better conducted by the tissue of the lung when dense than when in its natural condition. Moreover, Dr. Skoda has set this matter to rest by the following simple experiment, which he usually performs in the presence of his class, and which any one may easily repeat.

If the ear be applied to a stethoscope placed successively on corresponding parts of a sound and then of a hepatized lung removed from the body, the voice of another person who speaks through a stethoscope placed upon the lung at an equal distance in both cases, will be heard somewhat more distinctly in the sound than in the hepatized lung; but the distinction is so insignificant, that, were the reverse the case, it would not account for the very marked difference in such a condition of the lungs in the living subject.

Consonance is a term adopted by Dr. Skoda to express a well known phenomenon; and it may be here properly explained.

A tense guitar string sounds in unison with a note produced in its vicinity, either by another musical instrument or by the voice. A tuning fork held in the air emits a much weaker sound than when placed upon a table or chest. The table or chest must increase the intensity of the sound by assuming the same vibrations as the tuning fork, or, in other words, by consonating with it. The note of a Jew's harp is scarcely perceptible when it is struck in the air, and it is heard much more distinctly when played in the mouth. Thus the air in the mouth must increase the sound of the Jew's harp, *i. e.* must consonate with it.

It sometimes happens that the voice is heard more strongly at the thorax than at the laynx, which in itself is sufficient to show that its strength is increased by means of consonance within the chest. The different degrees of the intensity of the voice heard at the thorax, may be explained by the different strength of the consonance within the chest. To ascertain these changes we must disover what it is within the chest that consonates with the voice, and by what circumstances the consonance is liable to be altered.

The voice, as it issues from the mouth, is composed of the sound formed at the larynx, and the consonating sounds produced at the pharynx, mouth, and nasal cavities. This is shown by the alteration the voice undergoes by the opening and shutting of the nostrils and mouth, while there is no change made in the larynx. The pitch of the voice is evidently fixed by the larynx alone, and the opening and shutting of the nostrils and mouth has no influence upon it; the articulation of the voice, however, and its timbre, depend upon the mouth and nostrils.

As it is certain that the air in the pharynx, mouth and nostrils, consonates with the sound formed in the larynx, there can be no doubt that the air in the trachea and bronchiæ may also be thrown into consonant vibrations with the sounds formed at the larynx. Hence it is the air in the chest, and not the parenchyma of the lungs, which consonates with the voice at the larynx, as the latter seems ill adapted for consonating, being neither stiff nor sufficiently tense. Those substances, such as air, tense strings, membranes, slips of wood, and tin plates, in which a musical sound is most readily produced, are most easily thrown into consonant vibrations.

Air can consonate only when confined within a circumscribed space. In the open air the human voice, and every other sound, is heard more feebly than in a room. The air confined within the box of a guitar, violin, piano, &c., consonates with the note struck on the strings, while the sound is not increased by the consonance of the external air. The strength of the consonance depends upon the size and form of the space in which the air is confined, and upon the properties of the walls which bound the space. It appears that the consonating sound of the inclosed air will be stronger the more perfectly the walls reflect the sounds which spread through the air. A space surrounded by solid walls produces the greatest consonance, while in a linen tent the sound is but little increased. The cause of the strengthening of sounds by the speaking trumpet is well known.

The deductions drawn from the physical principles just referred to may be used in explaining the consonance of the voice in the chest. The air in the trachea and bronchia can consonate with the voice in as far as their walls resemble the walls of the larynx, mouth, and nasal cavities, in their power of reflecting sound. In the trachea, the walls of which consist of cartilage, the voice consonates almost as strongly as it sounds in the larynx. In the two branches also into which the trachea divides the consonance must be nearly as perfect. On the entrance of the bronchia into the parenchyma of the lung they have no longer cartilaginous rings, but merely thin irregular plates of cartilage interspersed in the fibrous tissue. As the bronchia ramify, these plants become smaller, thinner, and less numerous, and at last disappear altogether, and the finest twigs of the bronchia consist merely of membranous canals. In the normal state of the parenchyma of the lung the air in the bronchia consonates less strongly with the voice than that in the trachea, in proportion to the smaller number of cartilages they contain. The conditions which increase the consonance of the voice in the air contained within the bronchia that ramify in the parenchyma of the lung are either that the walls of the bronchia have become cartilaginous, or if still membranous, very thick, or that the surrounding tissue of the lungs has become devoid of air;—in all these conditions the walls reflect the sound more strongly than the membranous walls of the normal bronchia;—and there must be no interruption of continuity between the air in the bronchia and that in the larynx. If the air in a confined space be thrown into either original or imported autophonous vibrations, which give rise to sound, the surrounding walls not unfrequently partake of the same vibrations, and they do this the more readily the less stiff and hard they are.

In this respect a portion of the small intestine represents pretty well the more membranous parts of the bronchia, and a portion of the heart and liver the hepatized lung. If a person speaks through a stethoscope placed on one end of a moderately inflated small intestine, consonant vibrations of the voice, in the air within the intestine, may be heard by another person listening through a stethoscope placed on the other end of the intestine. If a layer of solid or fluid substance be interposed between the mouth of the stethoscope and the intestine, as, for example, a piece of liver or of intestine filled with water, the sound is heard very indistinctly, and not at all if the thickness of the interposed substance reaches half an inch.

If a passage be bored in the liver, so as not completely to pierce it through, and this be spoken into by means of a stethoscope accurately fitted into the entrance of it, the voice may be heard along the whole length of the passage, and for a considerable distance on each side, through a stethoscope placed over it, so strong, that it by far exceeds in intensity the voice proceeding from the mouth of the speaker, which is by the free air.—*Braithwaite's Retrospect, No.* 4, and *Edinburgh Med. and Surg. Journ.* July, 1841.

